# Remote delivery of congenital cardiac magnetic resonance imaging services, a unique telemedicine model

**DOI:** 10.1186/1532-429X-17-S1-P210

**Published:** 2015-02-03

**Authors:** Ruchira Garg, Arnel Sevilla, Ross Garberich, Fleishman Craig

**Affiliations:** Division of Pediatric Cardiology, Department of Pediatrics and The Heart Institute, Cedars-Sinai Medical Center, Los Angeles, CA USA; Radiology, Arnold Palmer Hospital for Children, Orlando, FL USA; Pediatrics, Division of Cardiology, Arnold Palmer Hospital for Children, Orlando, FL USA; Research, Minneapolis Heart Institute Foundation at Abbott Northwestern Hospital, MN Kragujevac, USA

## Background

Cardiac MRI is increasingly utilized in patients with congenital heart disease; however, the expertise to perform and interpret these studies is not universally available, despite an increasing population of congenital heart survivors. This retrospective analysis describes our experience providing CMRI services on-site versus over a distance of 250 miles.

## Methods

**Figure 1:**
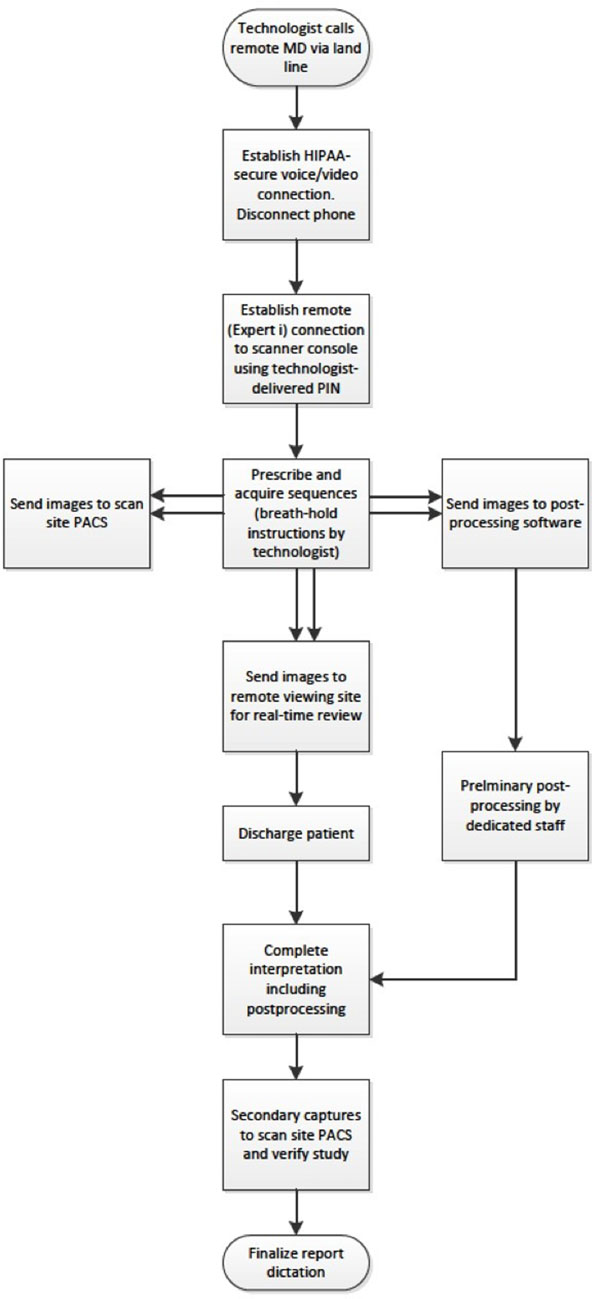
Figure 1

Our technique utilized the syngo Expert-i (Siemens) remote control software. Our configuration included a T3, high speed dedicated line with secure communication to achieve remote control of the scanner console without lag, immediate transfer of DICOM images, and to support secure voice and video over internet (VOIP). The remote site utilized a standard hospital-issued PC with a graphics card upgrade to install the Expert-i and VOIP software. The local site installed VOIP software on a laptop with built-in webcam. Figure 1 demonstrates the workflow for remote scan acquisition, image transfer, post-processing and completion of a study report.

We performed a retrospective descriptive analysis of our experience providing congenital cardiac MRI services both locally and from a remote location using the same physician providers.

## Results

Patient demographics and scan details are listed in Table 1. There were 83 "local" scans with both physician and patient on-site compared with 91 scans controlled by a physician geographically remote from the patients. The patients were well-matched for age, sex, study duration, scan type and history of prior cardiac surgery or intervention. There was no difference in use of deep sedation or diazepam for anxiolysis, or use of atropine for arrhythmia suppression. There were 2 minor events: A 23 hour observation was initiated by anesthesia in the local period after deep sedation in an infant with chronic lung disease, and a single patient experienced emesis after gadolinium administration in the remote period. There were no patient safety issues and there was satisfaction on the part of the referring physicians who were able to obtain more timely studies, as well as the remote-scanning physicians who had a workflow comparable with the local scans, but no lost travel time.

**Table 1:** Patient demographics and scan details.

	Local Scan Period	Remote Scan Period	P-Value
**DATES**	05/06 to 01/10	02/10 to 04/12	
**Number of patients**	83	91	
**Age (years), median (25** ^**th**^ **, 75** ^**th**^ **percentile)**	9.8 (3.5, 17.3)	10.6 (5.6, 16.8)	0.48
**Patient age < 6 years, (%)**	30 (36.1)	25 (27.5)	0.22
**Patient age = 6years, (%)**	53 (63.9)	66 (72.5)	0.22
**Male, (%)**	44 (53.0)	56 (61.5)	0.26
**Inpatients, (%)**	4 (5.2)	5 (5.7)	0.56
**Anesthesia-directed sedation, (%)**	39 (47.0)	30 (33.0)	0.059
**Diazepam, oral, (%)**	7 (8.4)	12 (13.2)	0.32
**Atropine, (%)**	3 (3.6)	4 (4.4)	0.79
**Prior cardiac surgery or catheter intervention, (%)**	57 (68.7)	54 (59.3)	0.20
**CMRI DURATION/COMPONENTS**			
**Study duration (minutes), mean(SD)**	58 ± 19	57 ± 19	0.76
**Study duration (minutes), median(25** ^**th**^ **, 75** ^**th**^ **percentile)**	57 (46, 73)	55 (42, 69)	0.50
**Scan Type**			
**Cardiac MRI only**	5 (6.0)	3 (3.3)	
**MRA chest only**	8 (9.6)	2 (2.2)	
**Cardiac MRI and MRA chest**	39 (47.0)	44 (48.4)	
**Cardiac MRI with MDE and MRA chest**	31 (37.4)	42 (46.2)	

* Assessed for significance using transformed variable

## Conclusions

This experience suggests that remote delivery of cardiac MRI services for the congenital heart population is feasible and can be done with comparable success and safety to a traditional "local" model. We also suggest the necessary configuration to provide such remote CMRI services with readily available hardware and software.

